# GC–MS metabolomics of French lettuce (*Lactuca Sativa* L. var *capitata*) leaves exposed to bisphenol A via the hydroponic media

**DOI:** 10.1007/s11306-024-02168-1

**Published:** 2024-09-21

**Authors:** Jerónimo Cabrera-Peralta, Araceli Peña-Alvarez

**Affiliations:** Universidad Nacional Autónoma de México, Av. Universidad, 3000 Mexico City, Mexico

**Keywords:** Metabolomics, Bisphenol A, Lettuce, Chemometrics, Gas chromatography–mass spectrometry

## Abstract

**Introduction:**

Bisphenol A (BPA), an organic compound used to produce polycarbonate plastics and epoxy resins, has become a ubiquitous contaminant due to its high-volume production and constant release to the environment. Plant metabolomics can trace the stress effects induced by environmental contaminants to the variation of specific metabolites, making it an alternative way to study pollutants toxicity to plants. Nevertheless, there is an important knowledge gap in metabolomics applications in this area.

**Objective:**

Evaluate the influence of BPA in French lettuce (*Lactuca Sativa* L. var *capitata*) leaves metabolic profile by gas chromatography coupled to mass spectrometry (GC–MS) using a hydroponic system.

**Methods:**

Lettuces were cultivated in the laboratory to minimize biological variation and were analyzed 55 days after sowing (considered the plant’s adult stage). Hexanoic and methanolic extracts with and without derivatization were prepared for each sample and analyzed by GC–MS.

**Results:**

The highest number of metabolites was obtained from the hexanoic extract, followed by the derivatized methanolic extract. Although no physical differences were observed between control and contaminated lettuce leaves, the multivariate analysis determined a statistically significant difference between their metabolic profiles. Pathway analysis of the most affected metabolites showed that galactose metabolism, starch and fructose metabolism and steroid biosynthesis were significantly affected by BPA exposure.

**Conclusions:**

The preparation of different extracts from the same sample permitted the determination of metabolites with different physicochemical properties. BPA alters the leaves energy and membrane metabolism, plant growth could be affected at higher concentrations and exposition times.

**Supplementary Information:**

The online version contains supplementary material available at 10.1007/s11306-024-02168-1.

## Introduction

Lettuce is one of the most economically important vegetables worldwide because its incorporation into the human diet brings multiple health benefits (Shatilov et al., 2019). Although lettuce is not usually considered a nutritious food due to its high water content (approximately 95%), it is a primary source of various nutrients such as minerals (principally Fe), vitamins (B_9_, C and E) and bioactive compounds (carotenoids and phenolic compounds). Also, despite its low lipid content, lettuce contains polyunsaturated fatty acids, such as linoleic and linolenic acids, which humans must obtain from diet (Kim et al., 2016). Additionally, although they are not considered nutrients, lettuces are rich in low-digestible carbohydrates like fiber, resistant starch and sugar alcohols, which are not absorbed in the small intestine but are necessary for adequate gastrointestinal functioning (Grabitske & Slavin, 2009).

Bisphenol A (BPA) is a xenobiotic organic compound used to produce polycarbonate plastics and epoxy resins, for elaborating thermal paper, food containers, water pipes and electronics (Zhang et al., 2019). BPA is transferred to the environment principally by direct contact of the products that contain it with residual or superficial waters (Catenza et al., 2021). Although wastewater treatment plants can remove approximately 62.5–99.6% of BPA, its high production volumes worldwide have led it to become a ubiquitous contaminant, having been determined in environmental matrices such as air, superficial water, soils, sediments, and dust; and in biological matrices like human blood, serum and maternal milk (Catenza et al., 2021; Chen et al., 2016). Furthermore, BPA is a known endocrine disruptor that adversely affects human reproduction and neural, immune and cardiovascular systems (Chen et al., 2016; Yamazaki et al., 2015). A review by Xiao et al. (2020) focused on reporting BPA toxicity to plants. It mentioned that the contaminant can alter their mineral absorption, photosynthesis and hormonal activity. It also stated that future research projects should further investigate the impact of BPA on plant biochemistry, a goal that can be achieved by metabolomics.

Among all the plant metabolomics applications, the evaluation of the effect of environmental contaminants on their metabolism is the least studied (Matich et al., 2019). Pollutants like plasticizers (Hurtado et al., 2017; Wang et al., 2018), pharmaceuticals (Picó et al., 2018), flame retardants (Chen et al., 2018), pesticides (Mahdavi et al., 2015; Pereira et al., 2014; Zhao et al., 2015) and nanoparticles (Chavez Soria et al., 2017; Ke et al., 2018; Li et al., 2018; McGehee et al., 2017; Salehi et al., 2018; Večeřová et al., 2016; Wu et al., 2017; Zahra et al., 2017; Zhao et al., 2016a, 2016b, 2017, 2018) have been evaluated in various matrices such as *Arabidopsis thaliana*, lettuce, reed, rice, cowpea, barley, tomato, bean, cucumber, corn and spinach. Despite the important knowledge gap in this field, metabolomics is a valuable tool for tracing the stress effects induced by the contaminants to the variation of specific metabolites that maintain the organism's homeostasis.

One of the principal difficulties for untargeted metabolomics is proposing an analytical method that can determine metabolites of various physicochemical properties (Castro-Puyana & Herrero, 2013; Gong et al., 2017). As the plant kingdom is estimated to have between 100,000 and 200,000 compounds (Hill & Roessner, 2013), there is no extraction procedure or instrumental technique able to determine simultaneously the complete plant metabolome. Thus, non-selective extractions are applied to determine the broadest possible fraction of metabolites, and the instrument is chosen based on its advantages and disadvantages for the analysis. Then, if a wider metabolic profile is required, extractions using different solvents or conditions and the application of more than one instrumental technique should be considered (Hill & Roessner, 2013).

Sample preparation is mistakenly disregarded in metabolomics, as its election affects the evaluated fraction of the metabolome and, hence, the biological interpretation of the study (Vuckovic, 2012). Most gas chromatography coupled to mass spectrometry (GC–MS) metabolomics protocols suggest preparing a single extract from each sample using either a single solvent or a mixture of them and then derivatizing (Fiehn, 2016; Mastrangelo et al., 2015; Rey-Stolle et al., 2022), which can result in the determination of metabolites with similar physicochemical properties. Regardless of being more time-consuming, the preparation of different extracts from the same sample, varying the solvents used for extraction and the derivatization procedures, allows the determination of a wider metabolic profile. Additionally, techniques alternative to solid–liquid extraction and liquid–liquid extraction that follow the principles of Green Chemistry should be used. Ultrasound-assisted extraction (UAE) can be a good choice for this matter, as it is a miniaturized sample preparation technique useful for tissue analysis due to cavitation capacity to affect membrane permeability, facilitating the analysis of intracellular content (Chemat et al., 2017; Mason, 2003).

The influence of BPA along with other 10 organic contaminants on lettuce leaves metabolic profile was previously determined by GC × GC–MS, concluding that these compounds altogether altered multiple metabolic pathways (Hurtado et al., 2017). As lettuces were cultivated in soil and exposed to a mix of pollutants, the study was able to evaluate the alteration of the metabolism simulating real crop conditions. Nevertheless, this system did not permit the determination of the individual effect of BPA on the leaves metabolic profile, and soil probably modified the concentration of the contaminants at which plants were originally exposed. Therefore, this project aimed to evaluate the influence of BPA in French lettuce (*Lactuca sativa* L. var. *capitata*) leaves metabolic profile by GC–MS using a hydroponic system.

## Material and methods

### Standards, reagents and materials

All organic solvents used in this work were reagent grade. Methanol, J.T. Baker (Trinidad and Tobago); hexanes, J.T. Baker (United States of America); ethyl acetate and pyridine, J.T. Baker (Mexico); methoxyamine hydrochloride, Sigma–Aldrich (United Kingdom) and MSTFA, Merck (Switzerland) were used for sample preparation. Deionized water acquired from a Millipore Direct-Q 3 UV system (Merck, USA) and sodium hydroxide (NaOH), J.T. Baker (Sweden) were used for hydroponic solution preparation. C7–C40 saturated alkanes standard, Supelco (United States of America) was injected for retention index (RI) calculation.

Bisphenol A (BPA) > 97%, Aldrich (Taiwan) was used for spiking the hydroponic media. For lettuce cultivation, the following materials were used: lettuce seeds acquired from a local store, hydroponic nutrients purchased as a powder from Hydro Environment (Mexico) and 600 W LED lamps, Surpson (USA) acquired from Amazon.

### Lettuce cultivation and sample pretreatment

Lettuces were cultivated hydroponically indoors using the floating root method. The hydroponic solution, renewed once a week at all stages of cultivation, was prepared by dissolving 15 g of hydroponic nutrients powder in 10 L of deionized water and adjusting pH to 6.0 with a 1 M NaOH aqueous solution. In a seedbed comprised of various individual cells, seeds were planted within a coconut fiber substrate moistened with hydroponic solution. The seedbed was suspended over a pool that contained 3 L of the hydroponic solution so that, when seeds germinated, their roots would grow towards the solution. LED lamps were used to eliminate the lettuce growth dependence because of weather conditions throughout the year, were hung 30 cm over the seedbed and set up in a daily program of 14 h of light and 10 h of dark. Eighteen seedlings were transferred to 2-L glass recipients three weeks after sowing, forming three groups of six lettuces each: control and spiked with BPA in the hydroponic solution at two different concentrations. The first spiked group was exposed to the contaminant at 5 ng/mL, as it was considered a representative concentration of BPA in superficial water and effluent proceeding from wastewater treatment plants in different countries (Catenza et al., 2021; Corrales et al., 2015; Muhamad et al., 2016); while the second group was exposed at 5 µg/mL, a significantly higher concentration than the first one. From this point on, the hydroponic solution was oxygenized seven times a day for 15 min every 2 h to promote lettuce growth; and 55 days after sowing, six leaves were harvested from each lettuce, immediately immersed in liquid nitrogen for metabolism quenching, freeze–dried for 48 h, homogenized in an agate mortar and stored in tightly closed vials inside a desiccator until their analysis.

### Sample preparation

#### Sample extracts

Each of the control and contaminated lettuce leaves samples were extracted by UAE using non-polar and polar solvents (hexane and methanol, respectively) and different post-extraction procedures to procure the determination of a wide metabolic profile. The four different sample extracts will be referred to as (1) hexanoic extract (HE), (2) methanolic extract (ME), (3) derivatized hexanoic extract (DHE) and (4) derivatized methanolic extract (DME). The general procedure was as follows.

#### Metabolites extraction and derivatization

##### Metabolites extraction

An amount of 5.0 mg of freeze–dried homogenized leaves was transferred to a 4 mL vial and 2 mL of extraction solvent (hexane or methanol) was added. Samples were subjected to UAE using an ultrasonic probe at 50% of wave amplitude for 3 or 7 min for hexane or methanol extraction, respectively. The solution was transferred to 2-mL Eppendorf tubes and centrifuged at 13,000 rpm for 5 min. The supernatant was transferred to a conical-bottom vial and evaporated with a gentle flow of nitrogen at 50 °C. If no derivatization procedure was applied, the solution was reconstituted with 150 µL of ethyl acetate, homogenized on a vortex agitator for 30 s and 1 µL of the solution was injected into the GC–MS system.

##### Derivatization

For samples subjected to the derivatization procedure, the vial containing the evaporated extract was covered in aluminum foil, 75 µL of methoxyamine hydrochloride at 20 mg/mL in pyridine were added, the solution was homogenized using a vortex agitator for 30 s and placed into an oven at 70 °C for 30 min. Then, 75 µL of MSTFA were added and, after vortex agitation for 30 s, the sample was placed into an oven at 70 °C for 30 min. The solution was cooled in a water bath at room temperature for 15 min and 1 µL was injected into the GC–MS system.

### GC–MS conditions

Gas chromatography coupled to mass spectrometry analyses was performed with an Agilent 6890N GC coupled to a 5973 MSD mass selective detector (Agilent Technologies, USA). A Zebron ZB-5 (30 m × 0.25 mm I.D., 0.25 μm F.T.) column was used (Phenomenex, USA). The oven temperature program was as follows: started at 60 °C for 1 min, then programmed at 10 °C/min to 310 °C and it was held for 5 min. Helium (99.999%, Praxair, Mexico) was used as carrier gas at 1 mL/min. The split/splitless injector temperature was at 250 °C with 1 μL as injection volume. Split (10:1) and splitless (1 min) modes were used for derivatized and non-derivatized extracts analysis, respectively. The MS ionization potential was 70 eV; the transfer line and ion source temperature were at 280 and 230 °C, respectively. A quadrupole mass analyzer was used, configured for SCAN acquisition from 40 to 550 m/z.

### Data processing workflow

Chromatograms were acquired using Agilent ChemStation software (version A.10.01). Chromatographic data processing (signal detection, deconvolution and integration) was achieved using AMDIS (version 2.73). Processing parameters were as follows: component width—12; resolution, sensitivity and shape requirements were set at medium; min. model peaks—5; min. S/N—20.0 (peak excluded if below); min. certain peaks—0.8; min. abundance—1.0; min. signal strength—20,000.0; weight limit—4. Peak areas were tabulated in Microsoft Excel, elaborating a data matrix for each sample extract (HE, DHE, ME and DME). Normalization, principal component analysis (PCA) and partial least squares-discriminant analysis (PLS-DA) of the four data matrixes in csv format were carried out in MetaboAnalyst 5.0. Identification of the most relevant metabolites was achieved by comparing their retention index and mass spectrum with those reported in the NIST20 mass spectral library. Pathway analysis of the identified metabolites was carried out in MetaboAnalyst 5.0.

### Metabolite extraction optimization

UAE was optimized separately for methanol and hexane extraction (applying the subsequent derivatization procedure) by analyzing control lettuce leaves using a 3^2^ factorial experiment design. The parameters evaluated were extraction time (3, 5 and 7 min) and wave amplitude (25, 50 and 75%) in a randomized order. As the obtained total ion chromatograms (TICs) presented many signals, 15 metabolites with the highest areas were used to represent the others for extraction optimization.

## Results and discussion

### Lettuces cultivation

Contrary to most metabolic studies reported in the literature, which determine the effect of contaminants on fruits and vegetables at an early stage of their growth, lettuces were analyzed 55 days after sowing, considered the adult stage of the plant. Thus, the observed metabolic modification could be associated with the alteration of its nutritional value when used for human consumption. Fig.S1 shows photographs of the cultivated lettuces on harvest day. No significant physical differences were observed between lettuces that belonged to control or contaminated groups.

### Metabolite extraction optimization

In both hexanoic and methanolic extraction, experiments using a wave amplitude of 75% caused solvent projections outside the vial, so results acquired using this condition were not considered in the data processing. Figs.S2 and S3 show the obtained multiple response optimization diagrams for both solvents. For hexanoic extraction, maximum desirability (configured to determine the condition which maximized the area for all the studied signals) was observed at either 50% and 3 min or at 25% and 7 min of wave amplitude and extraction time, respectively. The first pair of conditions were chosen as optimum to keep analysis time to a minimum. On the other hand, for methanolic extraction, maximum desirability was obtained at 50% of wave amplitude and either 5 or 7 min of extraction time. As the optimization only considered the 15 signals with the highest area and the TIC obtained with methanolic extraction was particularly complex, a higher extraction time was chosen to procure the reliable extraction of all the other metabolites. Therefore, the optimum conditions for methanolic extraction were 50% wave amplitude and 7 min.

### Metabolic profile obtained from the different sample extracts

**Fig. 1:**
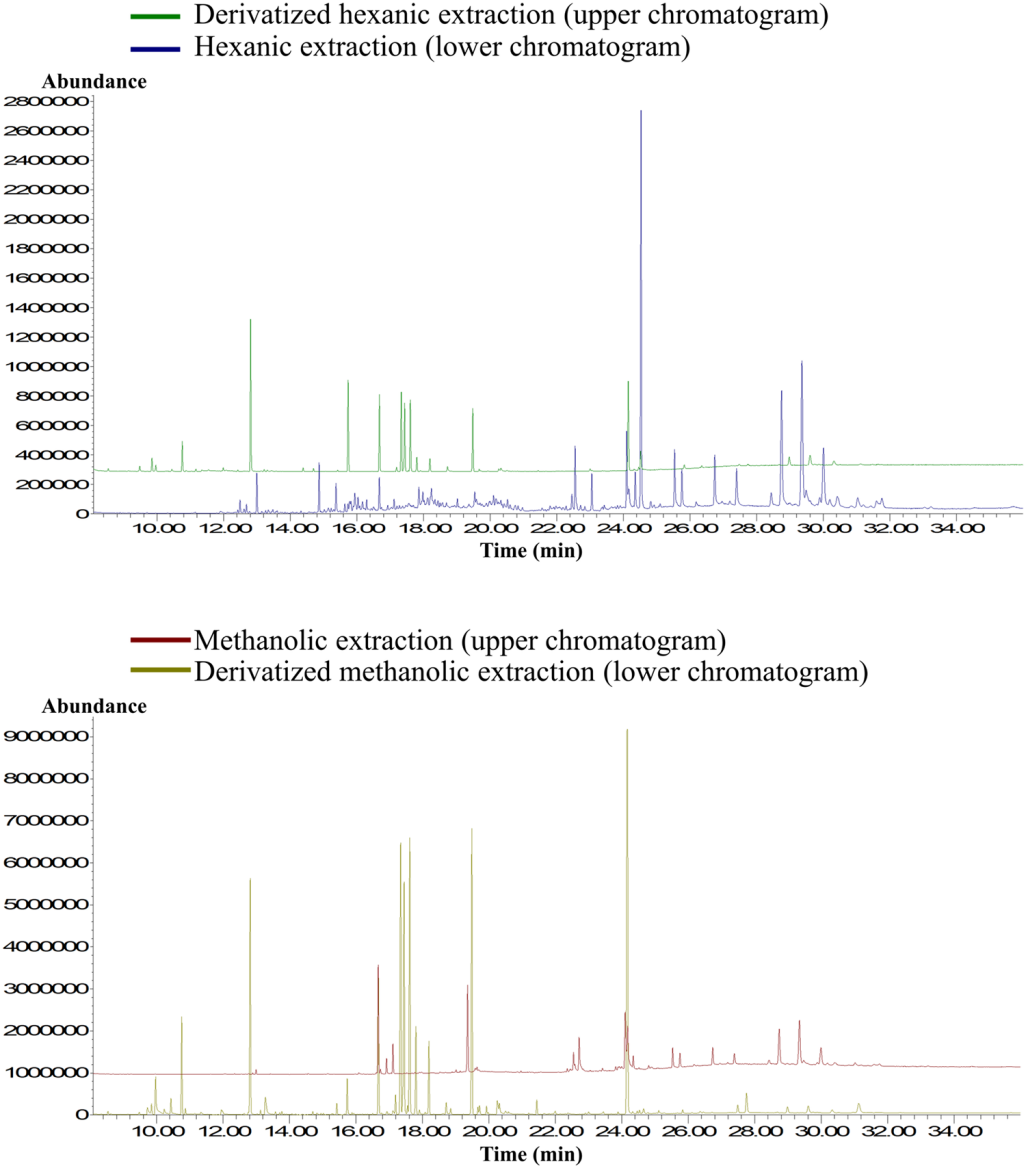
TICs of the analysis of the four different control lettuce leaves extracts by UAE-GC–MS. Chromatographic conditions described in Sect. 2.4

To give an overview of the differences between the metabolic profile obtained from the four different sample extracts, Fig. 1 shows an overlay of total ion chromatograms (TICs) acquired from control lettuces using each extract type. All the profiles were significantly different from one another, in general, the number of peaks and their intensities varied depending on the applied procedure. The same behavior was observed when comparing the metabolic profile obtained through each extract type of the contaminated lettuces at 5 ng/mL and 5 µg/mL. The analysis of four different extracts from the same sample has the advantage of determining a greater variety of metabolites.

The hexanoic extract permitted the extraction of 136 compounds, being the greatest number among all the different conditions. This was expected, as hexane mainly extracts non-polar volatile compounds that are most suitable for GC–MS analysis. On the other hand, the derivatized hexanoic extract permitted the extraction of 65 compounds, probably because the heat applied during the derivatization procedure led to the loss of the volatile metabolites in the solution. The methanolic extract was able to extract only 55 compounds, as methanol mainly extracts polar analytes that are then discriminated in the GC injector. However, the number of extracted compounds increased to 121 in the derivatized methanolic extract, as its derivatization procedure made possible the analysis of many polar analytes with low volatility.

### Multivariate analysis and metabolite identification

**Fig. 2:**
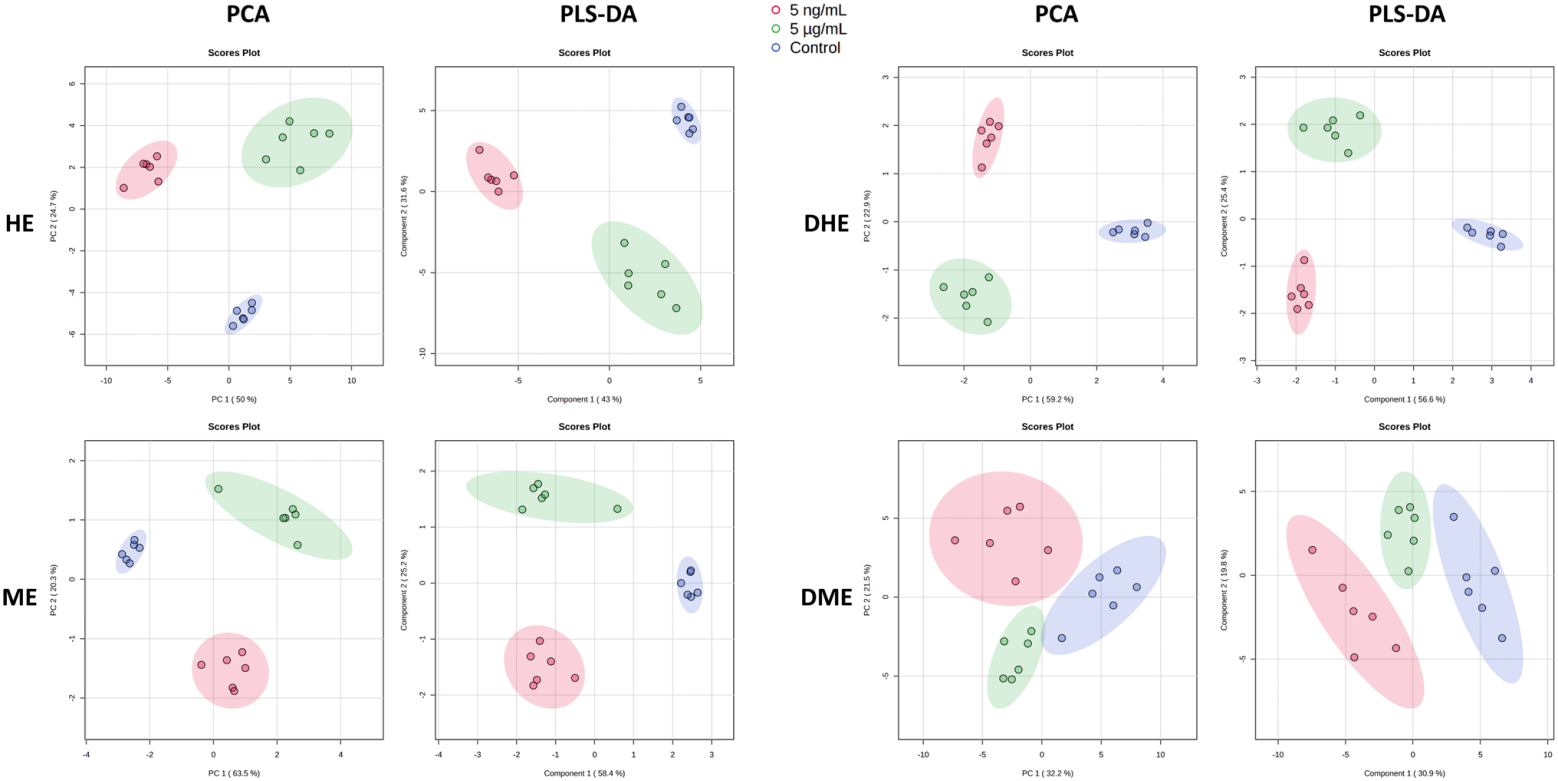
PCA and PLS-DA score graphics obtained from the analysis of the four elaborated data matrices. Blue: control; red: 5 ng/mL and green: 5 µg/mL

Figure 2 shows the PCA and PLS-DA score graphics obtained from the analysis of the four data matrices, elaborated from the GC–MS results of control and contaminated leaves analysis using each extract type. Separation between different groups of samples was observed in each plot, which demonstrated that both multivariate approaches detected a statistically significant difference between the leaves’ metabolic profile of control and exposed at 5 ng/mL and 5 µg/mL lettuces. PLS-DA fivefold cross-validation was performed, obtaining Q^2^ values above 0.8300 for all cases, which suggested that the model presented a good predictive ability.

Variable importance in projection (VIP) values obtained from the PLS-DA were used to determine which metabolites were mostly affected by BPA presence in the hydroponic media. Signals with VIP values > 1 were considered relevant for metabolic differentiation between sample groups (Chong & Jun, 2005). The 15 signals with the highest VIP values, which also met this criterion, were identified by matching their deconvoluted mass spectra and calculated RI with those contained in the NIST20 library (Tables 1, 2). A metabolite was considered as confidently identified when the resulting match factor and RI percentual difference were ≥ 600 and ≤ 5%, respectively, corresponding to a putatively annotated compound, or level 2 identification, according to the Metabolomics Standard Initiative (MSI). A total of 42 different metabolites were identified (Table S1), including organic and inorganic acids, sterols, terpenes, phenols, lactones, carbohydrates, glycerides, amino acids and long-chained alkanes, alkenes, alcohols, ethers and esters. Although some metabolites were detected using different sample extracts, most of them were only detected when a specific extraction solvent and post-extraction procedure were applied, reinforcing the importance of the method conditions on the metabolic profile to be determined.

**Table 1:** Identified compounds determined in the hexanoic extracts

Identified compound	VIP	RI (experimental)	RI (library)	%∆RI	Match factor	Identification level*
Hexanoic extract						
Docosanol	2.88	2493.8	2496	0.09	965	2
γ-sitosterol	2.67	3368.2	3321	1.40	949	2
Tetracosanol	2.59	2698.5	2698	0.02	901	2
(3β,5α)-stigmast-7-en-3-ol	2.52	3424.4	3377	1.38	902	2
Hexacosanol	2.52	2902.7	2857	1.57	946	2
Stigmasterol	2.44	3310.3	3249	1.85	927	2
2-methyl-octadecane	1.81	1929.6	1863	3.45	790	2
Neophytadiene	1.63	1841.5	1838	0.19	924	2
γ-tocopherol	1.63	3076.2	3057	0.62	943	2
1-hexacosene	1.51	2595.6	2595	0.02	662	2
1,3,5-tri-2-propenyl-1,3,5-triazyn-2,4,6(1H,3H,5H)-trione	1.50	1674.3	1661	0.79	932	2
3,7,11,15-tetramethyl-1-hexadecanol	1.48	2080.1	2074	0.29	899	2
Eicosyloctyl ether	1.30	2799.3	2860	2.17	830	2
(3β,24Z)-stigmasta-5,24(28)-dien-3-ol	1.23	3384.1	3343	1.21	788	2
Methyl 2-methylhexacosanoate	1.23	2998.6	2908**	3.02	626	2
Derivatized hexanoic extract						
2TMS (E)-Erithrono-1,4-lactone	2.45	1384.6	1400**	1.11	885	2
5TMS gulose	2.37	2062.0	1970**	4.46	684	2
Not identified	2.20	2282.0	N/A	N/A	N/A	4
TMS campesterol	2.12	3303.9	3252	1.57	716	2
Not identified	2.09	1964.2	N/A	N/A	N/A	4
2TMS 1-monopalmitine	1.82	2599.3	2606	0.26	855	2
2TMS 1-monolinoleine	1.75	2769.2	2743	0.95	784	2
TMS 2,4-di-tert-butylphenol	1.60	1553.6	1549	0.30	927	2
2TMS 1-linolenoyl glycerol	1.59	2779.1	2753	0.94	800	2
Docosanoic acid methyl ester	1.47	2530.8	2528	0.11	871	2
TMS myristic acid	1.42	1851.1	1850	0.06	814	2
1,3,5-tri-2-propenyl-1,3,5-triazyn-2,4,6(1H,3H,5H)-trione	1.41	1674.6	1661	0.81	889	2
TMS docosanoic acid	1.38	2641.6	2636	0.21	861	2
Tetracosanoic acid methyl ester	1.28	2732.1	2730	0.08	876	2
TMS hexacosanoic acid	1.25	3036.9	3036	0.03	847	2

^*^Identification level for metabolites according to Metabolomics Standard Initiative (MSI)

^**^Retention indexes correspond to predicted values reported by the NIST20 library

*N/A* not applicable, *TMS* Trimethylsilyl derivative

**Table 2:** Identified compounds determined in the methanolic extracts

Identified compound	VIP	RI (experimental)	RI (library)	%∆RI	Match factor	Identification level*
Methanolic extract						
Tricosanoic acid methyl ester	4.10	2631.1	2628	0.12	748	2
Pentacosanoic acid methyl ester	2.69	2832.3	2823	0.33	816	2
Docosanoic acid methyl ester	2.03	2530.2	2528	0.09	935	2
Linolenic acid methyl ester	1.77	2105.8	2099	0.32	850	2
Not identified	1.71	2155.0	N/A	N/A	N/A	4
Octacosanoic acid methyl ester	1.70	3133.8	3126	0.25	903	2
Not identified	1.61	1608.8	N/A	N/A	N/A	4
2,4-di-tert-butylphenol	1.61	1515.0	1514	0.07	905	2
Triacontanoic acid methyl ester	1.59	3334.2	3323	0.36	837	2
Tetracosanoic acid methyl ester	1.50	2732	2730	0.07	946	2
(3β,5α,24S)- stigmast-7-en-3-ol	1.39	3423.3	3353**	2.05	913	2
Hexacosanoic acid methyl ester	1.28	2934	2935	0.03	922	2
3,7,11,15-tetramethyl-1-hexadecanol	1.06	2079.9	2074	0.28	848	2
Derivatized methanolic extract						
8TMS sucrose	3.65	2707.3	2709	0.06	963	2
6TMS myo-inositol	2.84	2131.4	2090	1.94	951	2
3TMS malic acid	2.45	1500.5	1497	0.23	955	2
6TMS scyllo-inositol	2.15	1993.4	1982**	0.57	878	2
2TMS valine	1.99	1224.6	1221	0.29	949	2
3TMS phosphoric acid	1.87	1286.3	1285	0.10	890	2
MO, 5TMS d-galactose	1.81	1928.1	1939	0.59	921	2
TMS de (3β,5α)-stigmast-7-en-3-ol	1.77	3450.2	3391	1.72	801	2
MO, 5TMS d-fructose	1.75	1907.0	1905	0.10	920	2
Not identified	1.69	1964.4	N/A	N/A	N/A	4
MO, 5TMS d-fructose (sin)	1.68	1916.9	1869	2.50	913	2
Tetracosanoic acid methyl ester	1.63	2732.9	2730	0.11	913	2
Hexacosanoic acid methyl ester	1.61	2934.6	2935	0.01	927	2
6TMS 2-phenylethyl 2-O-β-D-xilopiranosyl-β-D-glucopiranoside	1.58	3204.6	3280	2.35	691	2
Not identified	1.56	2758.7	N/A	N/A	N/A	4

^*^Identification level for metabolites according to Metabolomics Standard Initiative (MSI)

^**^Retention indexes correspond to predicted values reported by the NIST20 library

*N/A* not applicable, *TMS* Trimethylsilyl derivative, *MO* Methyloxime derivative

### Preliminary prediction of BPA biological impact on lettuce leaves

**Fig. 3:**
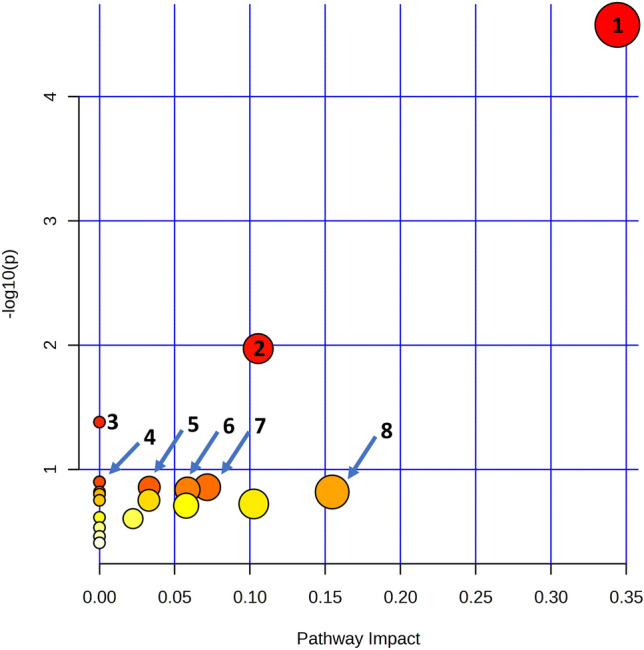
Pathway analysis diagram for the prediction of the influence of BPA on lettuce leaves metabolism. The eight most impacted pathways were: 1. Galactose metabolism; 2. Starch and sucrose metabolism; 3. Steroid biosynthesis; 4. Cutine, suberine and wax biosynthesis; 5. Citrate cycle; 6. Carbon fixation in photosynthetic organisms; 7. Fructose and mannose metabolism; 8. Pyruvate metabolism

Pathway analysis was performed on MetaboAnalyst based on the Kyoto Encyclopedia of Genes and Genomes (KEGG) pathway library for *Arabidopsis thaliana* to predict the biological impact of the identified metabolite variation on lettuce metabolism. Of the 42 identified compounds, only 16 were contained in the database (Table S2). Figure 3 shows the resulting plot in which various pathways are represented as circles. As their color changed from white to colored, the metabolites statistical significance to the affected pathway increased. Also, as the circle size augmented, the metabolites were positioned in a more crucial place in the pathway (e.g., a source or convergence of multiple nodes), making their variation more critical. The eight pathways that were statistically more affected (with higher − log(P) values) are also indicated in Fig. 3.

**Fig. 4:**
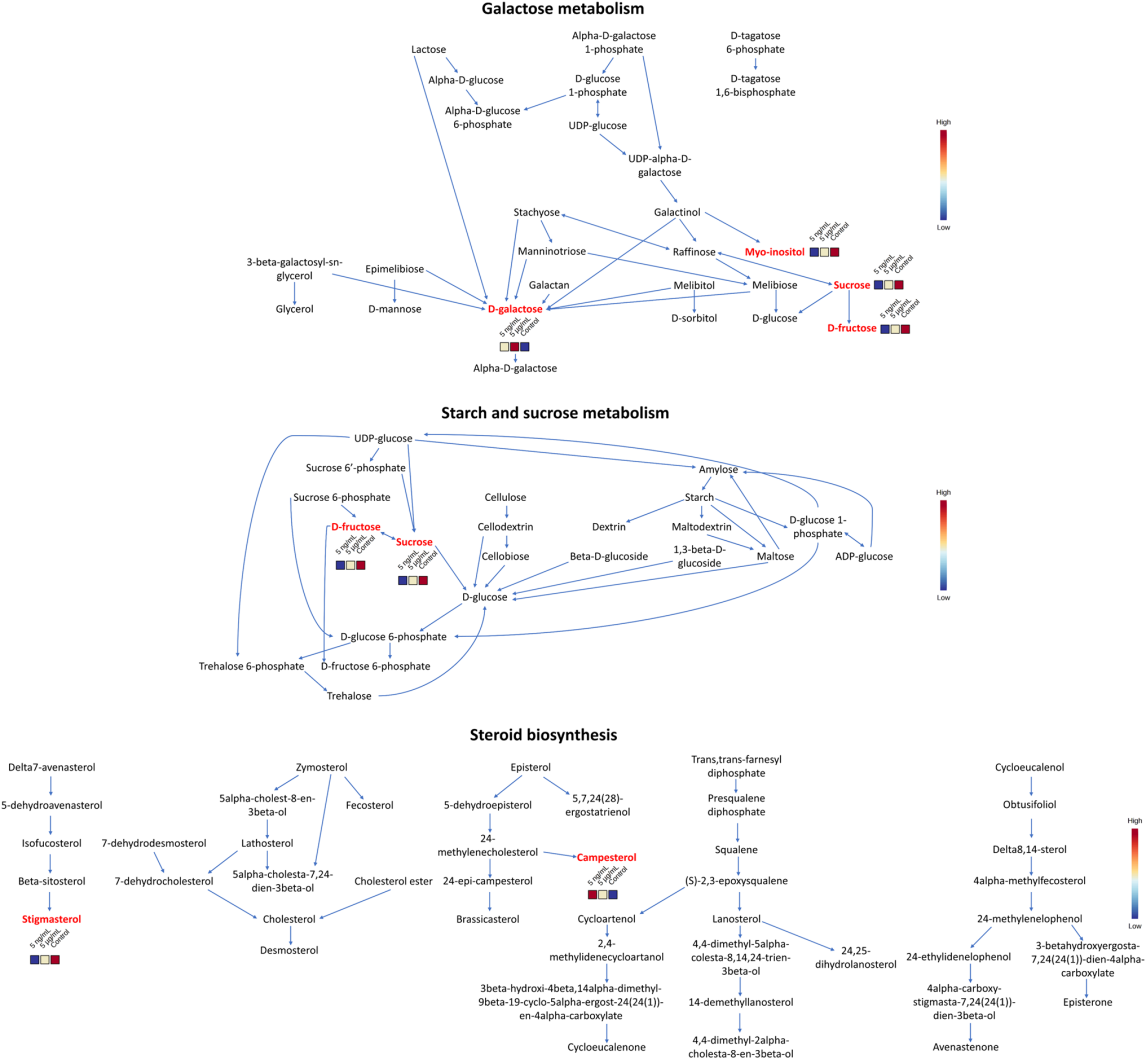
Detailed view of galactose metabolism, starch and sucrose metabolism and steroid biosynthesis. Metabolites marked in red were identified in the present study. Blue, cream and red colors correspond to a low, mid or high intensity of the metabolite in the leaves, respectively

The galactose metabolism was the most significantly affected and the one with the highest pathway impact because four significantly modified metabolites participate in the pathway (Fig. 4), and due to galactose being the conversion of several nodes (referred to as a “bottleneck”). Notably, the galactose metabolism, starch and sucrose metabolism and the steroid biosynthesis pathways were affected by the variation of more than one of the significantly impacted metabolites (Fig. 4), increasing the relevance of their alteration.

As shown in Fig. 4, galactose intensity in leaves increased as samples were exposed to higher concentrations of BPA. Oligosaccharides from the raffinose family (composed of sucrose and one or more galactose units) are accumulated in plants under stress conditions to serve as alternative carbohydrate sources for the cell (ElSayed et al., 2013). The rupture of these oligosaccharides produces free galactose, which is in turn transformed into α-d-galactose (as seen in Fig. 4) or glucose by galactolysis, being the latter monosaccharide useful for energy generation (Althammer et al., 2020). Therefore, lettuce exposure to BPA could block the enzymes responsible for galactose recycling. It is noteworthy that free galactose can be toxic to plants even at low concentrations (Althammer et al., 2020), so the increase in the intensity of this metabolite in leaves, principally when exposed to BPA at 5 µg/mL, could be a sign that lettuces were close to a significant metabolic alteration.

In comparison with the control group, sucrose intensity decreased in leaves of lettuces exposed to BPA at 5 ng/mL (Fig. 4). Various plants majorly use sucrose to transport photoassimilates from chloroplasts to sink tissues via the phloem. During the daytime, carbohydrates are continuously produced and temporally stored as starch in the chloroplasts. At night, they are transformed into sucrose and transported to non-photosynthetic cells (Hans-Walter, 2005). Therefore, the two possible reasons for this decrease in sucrose intensity are the diminishment of photosynthetic activity or an increase in sucrose migration from the leaves. As a blockage in photosynthesis would have directly impacted plants' growth and, as showed in Fig.S1, no apparent physical changes were observed between control and contaminated groups, there was probably an increase in sucrose transfer to other organs, augmenting their energy metabolism to counter the stress induced by BPA. This theory is reinforced by the fact that sucrose can also function as an osmoprotectant with a high radical-scavenging capacity (Van den Ende & Valluru, 2009). As BPA stress in several plants has proven to induce the formation of reactive oxygen species (ROS) (Xiao et al., 2020), probably sucrose in leaves was also used for ROS scavenging, or its transference to other organs was also prioritized to counter the formation of this high-toxicity species in other plants organs.

Sucrose intensity in leaves of lettuces exposed to BPA at 5 µg/mL also diminished compared to the control group, but it was higher than in lettuces exposed to the contaminant at 5 ng/mL (Fig. 4). This may suggest that the metabolic modification that allowed the lettuces to adapt to the stress induced by BPA at 5 ng/mL was altered when the contaminant concentration increased to 5 µg/mL. However, these conditions were still not enough to cause significant modifications to the leaves’ growth. This hypothesis can be confirmed through the work of Ferrara et al. (2006), as they observed that exposing lettuces to BPA at higher concentrations than 10 μg/mL resulted in decreased plant growth. Therefore, BPA at 5 µg/mL could be starting to affect the photosynthetic activity of the leaves and, at higher contaminant concentrations or exposition times, it could hinder their growth. This shows that lettuces exposed to the contaminant at 5 µg/mL were indeed close to a significant metabolic alteration, just as discussed earlier. It is important to note that the behavior observed for the variation of sucrose intensity in control and contaminated leaves was also shown for other metabolites (Figs.S4–S7) and could be explained by the same hypothesis.

The behavior of fructose intensity in control and contaminated leaves was the same as for sucrose (Fig. 4), probably because it is one of the breakdown products of the disaccharide. Also, fructose is stored in cells as fructans (composed of sucrose and one or more fructose units) to serve as secondary carbohydrate storage. Fructans are produced and stored in vacuoles, and approximately 80% of the leaf volume is composed of these organelles (Hans-Walter, 2005), so fructose can be an abundant metabolite in leaves. Therefore, the decrease in this monosaccharide intensity in leaves of lettuces exposed to BPA at 5 ng/mg could be due to this metabolite transfer to no-photosynthetic organs to counter the stress induced by the contaminant. Furthermore, fructose participates in the formation of secondary metabolites that act as ROS scavengers (Rosa et al., 2009). Thus, its intensity may have also decreased to produce scavengers to diminish the concentration of these toxic species in the leaves or due to its transference to other organs to serve for the same purpose.

Myo-inositol intensity in control and contaminated leaves also followed the same trend as sucrose (Fig. 4), probably because glucose, the other sucrose breakdown product, is used to produce this metabolite. Myo-inositol is used to form a great variety of compounds that are useful for diverse plants' biochemical functions, being its conformational isomers an example (Siracusa et al., 2022). Scyllo-inositol, which was also identified in this study, is a stereoisomer of myo-inositol, so its intensity in leaves presented the same behavior as its parent compound (Fig.S7). The intensity of both metabolites in lettuces exposed to BPA was probably modified because O-methyl derivatives of myo-inositol and its isomers participate in plant stress-related responses (Loewus & Murthy, 2000). The decrease in the intensity of myo-inositol in the leaves could have also impacted the formation of galactinol, a compound composed of this metabolite and galactose, which is needed for the formation of the oligosaccharides of the raffinose family (Loewus & Murthy, 2000). The hindrance in the formation of these carbohydrates probably caused the accumulation of galactose in the leaves discussed earlier.

The intensity of stigmasterol and campesterol, two sterols that are end products in the steroid biosynthesis pathway (Fig. 4), was significantly modified due to the lettuces’ exposure to BPA. Stigmasterol intensity in leaves of lettuces exposed to the contaminant decreased in comparison to the control group (Fig. 4). Stigmasterol is a component of mitochondria, endoplasmic reticulum and plasmatic membranes, whose concentration can be modified to alter their fluidity, permitting the cells to adapt to biotic and abiotic stress (Ahammed et al., 2020; Schaller, 2003). On the other hand, in comparison with lettuces of the control group, campesterol intensity in leaves of contaminated lettuces increased (Fig. 4). Aside from a membrane constituent, campesterol is an important precursor for the synthesis of brassinosteroids, which are phytohormones that are principally related to plants growth and have demonstrated to improve plants photosynthesis efficiency under stress conditions (Aboobucker & Suza, 2019; Schaller, 2003). As lettuces from control and BPA-exposed groups did not show significant differences in their growth, campesterol intensity probably increased in contaminated lettuces to promote brassinosteroids production, ensuring adequate lettuce growth.

Malic acid is another of the identified metabolites that also perform relevant functions in plants. Its intensity in leaves increased in the lettuce group exposed to BPA at 5 ng/mL. However, it decreased in the lettuces exposed to the contaminant at 5 µg/mL (Fig.S7). Malic acid is an intermediary of the citrate cycle, which is crucial to plants energy generation (Zhang & Fernie, 2023). This behavior further proves that lettuces exposed to BPA at 5 µg/mL were close to a metabolic alteration that could have impacted plants growth. The citrate cycle can use different metabolites as precursors of its cycle intermediates to adapt to environmental changes. Valine, which was also identified in this study, is a branched-chain amino acid used to replenish succinyl-CoA reserves (Raussell & Taegtmeyer, 2013), so its intensity in contaminated lettuces leaves may have increased to maintain the citrate cycle stabilization (Fig.S7). γ-tocopherol intensity in leaves decreased in lettuces exposed to BPA at 5 ng/mL and increased in lettuces exposed at 5 µg/mL (Fig.S4). Tocopherols serve as antioxidants, membrane stabilization agents and participate in cyclic electron transport in photosystem II (Munné-Bosch & Falk, 2004), so its modification further confirms that BPA exposure significantly affects leaves general energy and membrane metabolism.

Lettuce leaves metabolic profile behavior due to BPA exposure agreed with literature reports of other plant matrixes subjected to this contaminant. Sarkar and Roy (2024) studied the metabolic variation of the aquatic fern *Azolla filiculoides* on a system spiked with BPA at 1 and 30 µg/mL. The evaluated exposition times for each concentration were 3 and 9 days. Physical damage to the plant was only observed at the higher concentration, but multivariate analysis determined a statistically significant difference between each of the evaluated conditions. They concluded that BPA diminished de activity of plants primary metabolism enhancing their secondary metabolism, which is responsible of ROS scavenging, to counter the formation of this toxic species. Xiao et al. (2019) evaluated the effect of BPA on soybean root cells mitochondria. Seedlings were exposed to the contaminant at 1.5, 6 and 17.2 µg/mL for 7 days. Membrane permeability was monitored along with levels of ROS, ATP and important enzymes for energy generation. The lower BPA dose caused almost no damage to the root tip, while the medium and higher doses caused significant root cell death. They reported that BPA induced ROS stress to the cell, damaging the membrane and inhibiting the function of energy-generating enzymes, which led to the significant decrease of ATP in the cell, promoting cell death.

The variation of lettuce leaves metabolic profile due to BPA exposure directly impacted their nutritional value. Specifically for low-digestible carbohydrates, fiber contents were probably affected by the modification of the identified mono and disaccharide intensity, starch metabolism was affected by the variation of sucrose and fructose intensity in leaves and sugar alcohols intensity (such as myo- and scyllo-inositol) was also modified. On the other hand, the variation of linolenic acid methyl ester, monopalmitine, monolinoleine and 1-linolenoyl glycerol intensity in the leaves suggests that their corresponding polyunsaturated fatty acids were also affected by the lettuce exposure to BPA.

## Conclusions

No significant physical differences were found between control lettuces and those exposed to BPA at any of the studied concentrations. However, their metabolic profile analysis showed a statistically significant difference. Galactose metabolism, starch and fructose metabolism, as well as steroid biosynthesis were affected by BPA exposure, mainly altering the leaves energy and membrane metabolism. The intensity modification of some identified metabolites suggested that lettuces exposed to BPA at 5 µg/mL were close to a significant alteration in their metabolism that could have impacted leaves’ growth. The extraction of metabolites using different solvents and implementing or avoiding derivatization allowed the determination of a greater variety of metabolites. Whether studied individually or in complex mixtures, plant metabolomics of environmental pollutants will gradually help understand their true effect on the environment.

## Supplementary Information

Below is the link to the electronic supplementary material.

Supplementary file1 (PDF 694 KB)

## Data Availability

No datasets were generated or analysed during the current study.
