# Supplementary material for: GC–MS metabolomics of French lettuce (Lactuca Sativa L. var capitata) leaves exposed to bisphenol A via the hydroponic media
Source: Metabolomics. 2024 Sep 21;20(5):106. doi: 10.1007/s11306-024-02168-1 (PMC11416399; doi:10.1007/s11306-024-02168-1)
Supplement: Supplementary file 1 — Supplementary file1 (PDF 694 KB) [file 11306_2024_2168_MOESM1_ESM.pdf]

GC-MS metabolomics of *Lactuca Sativa* L. leaves exposed to Bisphenol A via the hydroponic media

Metabolomics

Jerónimo Cabrera-Peralta [a], Araceli Peña-Alvarez [a]*

[a] Universidad Nacional Autónoma de México, Av. Universidad, 3000, CDMX, Mexico
e-mail address: arpeal@unam.mx

Supplementary material

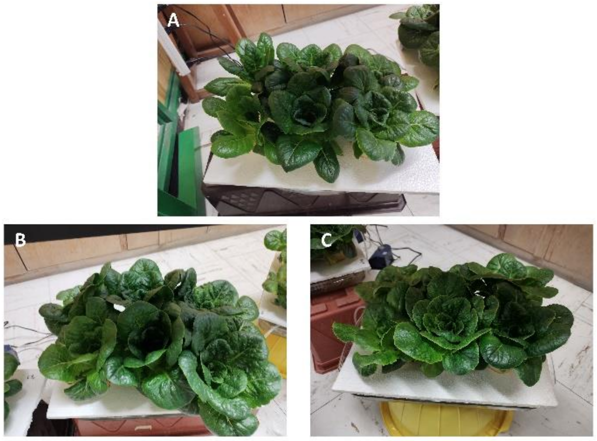

Fig.S1 Cultivated hydroponic lettuces 55 days after sowing. A. Control group. Lettuces exposed to BPA at: B. 5 ng/mL and C. 5 µg/mL

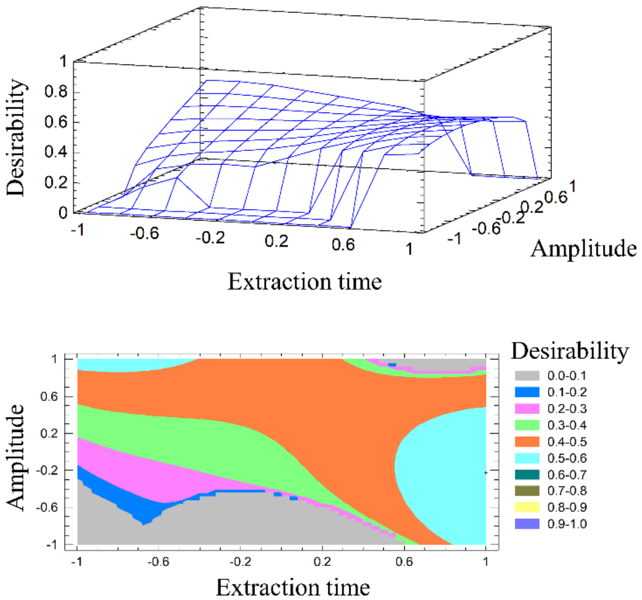

Fig.S2 Multiple response optimization diagram obtained in the optimization of the hexanic extract (DHE) analysis conditions

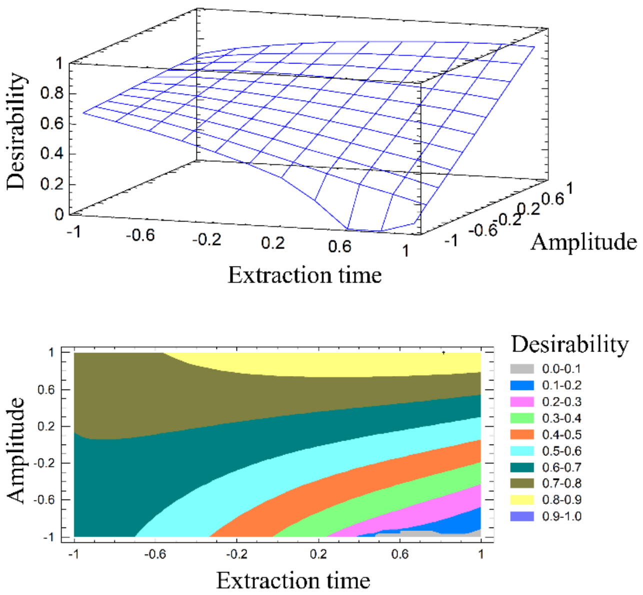

Fig.S3 Multiple response optimization diagram obtained in the optimization of the methanolic extract (DME) analysis conditions

Table S1 List of the 42 identified metabolites in the different sample extracts

**Sample preparation method**	**Metabolite**
HE	Docosanol
HE	γ-sitosterol
HE	Tetracosanol
HE, ME, DME	(3β,5α)-stigmast-7-en-3-ol
HE	Hexacosanol
HE	Stigmasterol
HE	2-methyl-octadecane
HE	Neophytadiene
HE	γ-tocopherol
HE	1-hexacosene
HE, DHE	1,3,5-tri-2-propenyl-1,3,5-triazyn-2,4,6(1H,3H,5H)-trione
HE, ME	3,7,11,15-tetramethyl-1-hexadecanol
HE	Eicosyloctyl ether
HE	Triethylen glycol di(2-ethylhexoate)
HE	(3β,24Z)-stigmasta-5,24(28)-dien-3-ol
ME	Tricosanoic acid methyl ester
ME	Pentacosanoic acid methyl ester
ME, DHE	Docosanoic acid methyl ester
ME	Linolenic acid methyl ester
ME	Octacosanoic acid methyl ester
ME, DHE	2,4-di-tert-butylphenol
ME	Triacontanoic acid methyl ester
ME, DHE, DME	Tetracosanoic acid methyl ester
ME, DME	Hexacosanoic acid methyl ester
DHE	(E)-erithrono-1,4-lactone
DHE	d-gulose
DHE	Campesterol
DHE	1-monopalmitine

Table S1 List of the 42 identified metabolites in the different sample extracts *(continued)*

Sample preparation method	Metabolite
DHE	1-monolinoleine
DHE	1-linolenoyl glycerol
DHE	Myristic acid
DHE	Docosanoic acid
DHE	Hexacosanoic acid
DME	Sucrose
DME	Myo-inositol
DME	Malic acid
DME	Scyllo-inositol
DME	Valine
DME	Phosphoric acid
DME	d-galactose
DME	d-fructose
DME	2-phenylethyl 2-O-β-D-xilopiranosyl-β-D-glucopiranoside

Table S2 Metabolites subjected to Pathway analysis

Number	Metabolite	KEGG ID
1	γ-sitosterol	C19654
2	(3β,5α)-stigmast-7-en-3-ol	C08839
3	Hexacosanol	C08381
4	Stigmasterol	C05442
5	γ-tocopherol	C02483
6	Campesterol	C01789
7	Myristic acid	C06424
8	Docosanoic acid	C08281
9	Sucrose	C00089
10	Myo-inositol	C00137
11	Malic acid	C00149
12	scyllo-inositol	C06153
13	Valine	C00183
14	Phosphoric acid	C00009
15	d-galactose	C00124
16	d-fructose	C00095

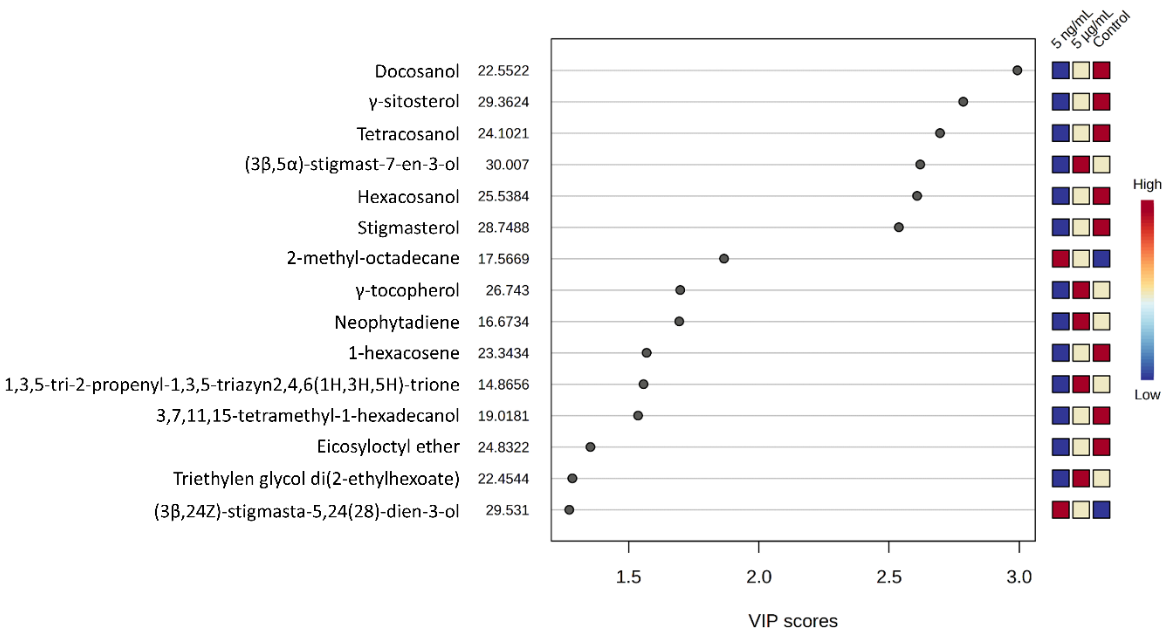

Fig.S4 VIP plot obtained from the PLS-DA of the HE results

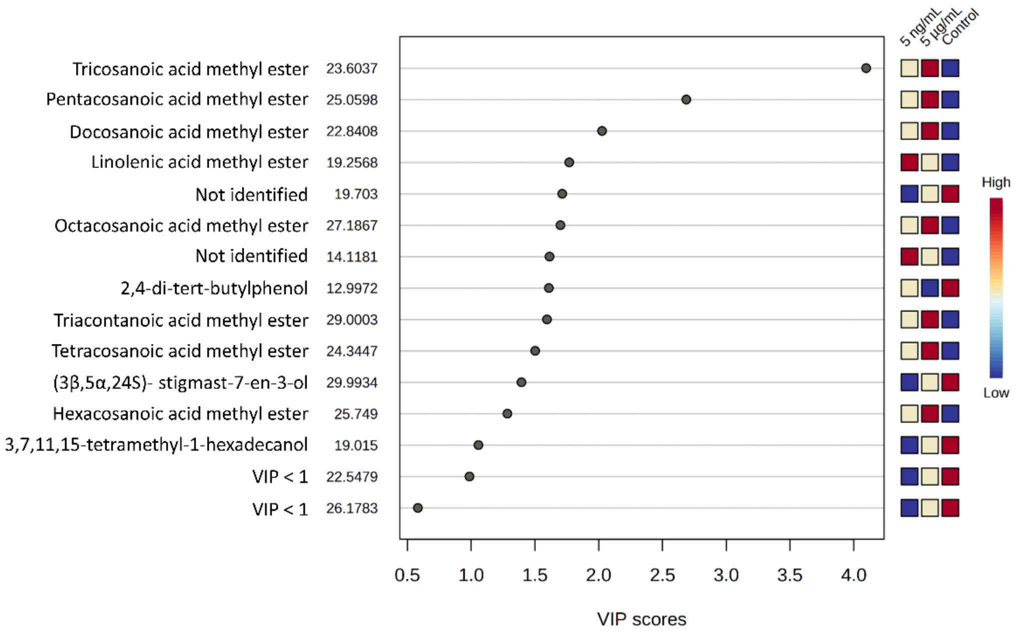

Fig.S5 VIP plot obtained from the PLS-DA of the ME results

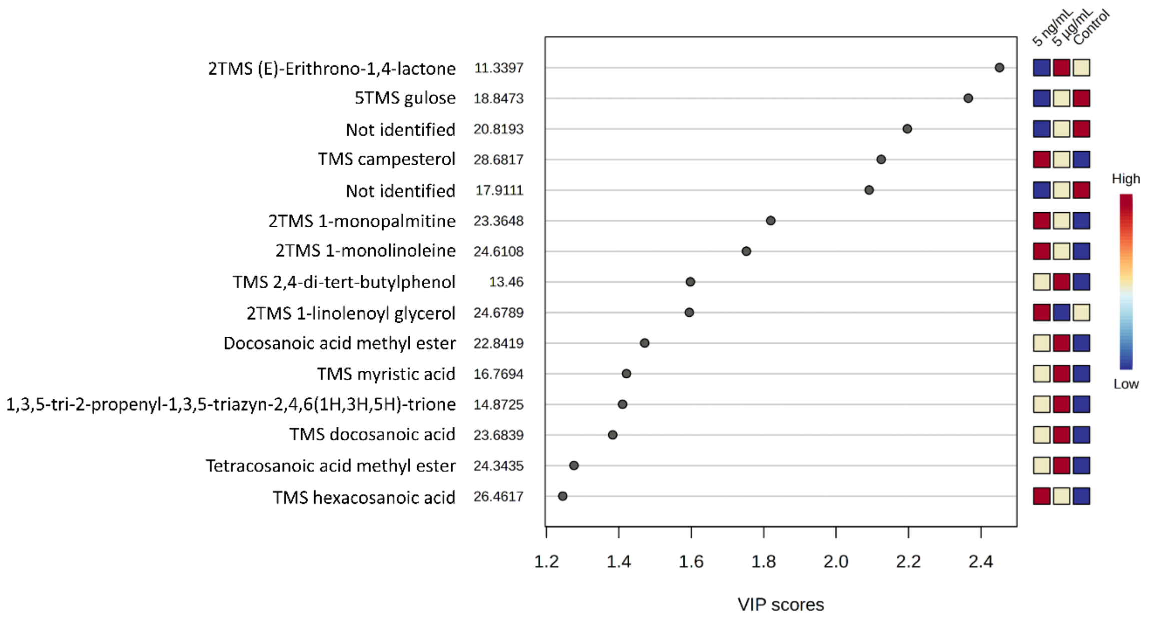

Fig.S6 VIP plot obtained from the PLS-DA of the DHE results

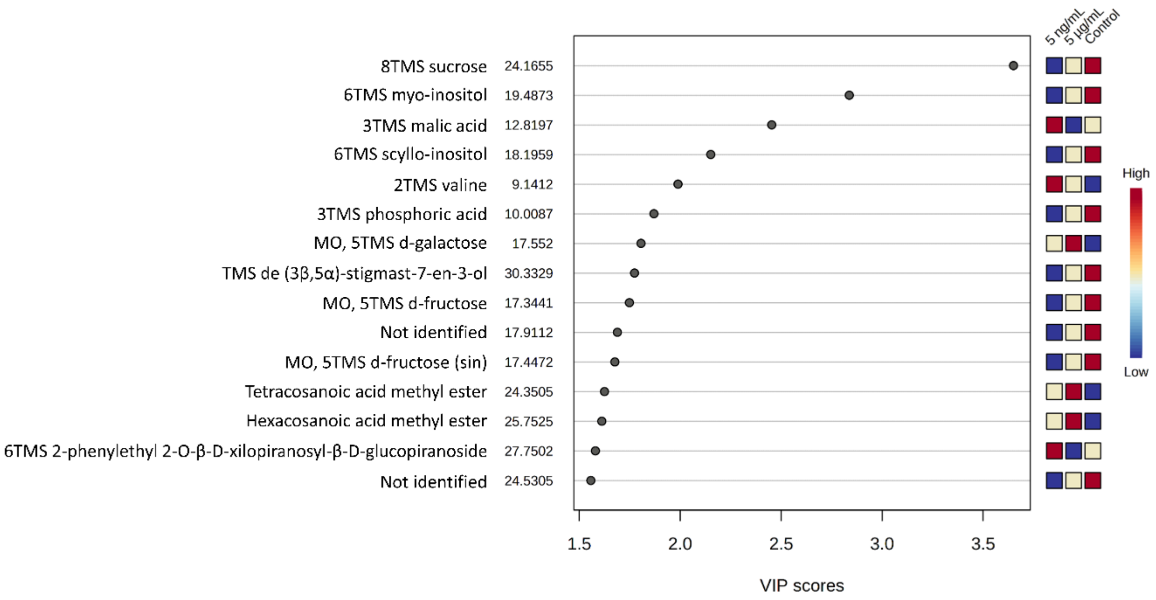

Fig.S7 VIP plot obtained from the PLS-DA of the DME results
